# Negative self-perception of hearing and depression in older adults: a population-based study

**DOI:** 10.11606/s1518-8787.2023057004675

**Published:** 2023-03-29

**Authors:** Karina Mary de Paiva, Alessandra Giannella Samelli, Pamela Lopes de Oliveira, Danúbia Hillesheim, Patrícia Haas, Paulo Adão de Medeiros, Eleonora d’Orsi

**Affiliations:** I Universidade Federal de Santa Catarina Departamento de Fonoaudiologia Florianópolis SC Brasil Universidade Federal de Santa Catarina. Departamento de Fonoaudiologia. Florianópolis, SC, Brasil; II Universidade de São Paulo Faculdade de Medicina Departamento de Fisioterapia, Fonoaudiologia e Terapia Ocupacional São Paulo SP Brasil Universidade de São Paulo. Faculdade de Medicina. Departamento de Fisioterapia, Fonoaudiologia e Terapia Ocupacional. São Paulo, SP, Brasil; III Universidade Federal de Santa Catarina Programa de Pós-Graduação em Saúde Coletiva Florianópolis SC Brasil Universidade Federal de Santa Catarina. Programa de Pós-Graduação em Saúde Coletiva. Florianópolis, SC, Brasil; IV Associação Nacional de Gerontologia Florianópolis SC Brasil Associação Nacional de Gerontologia. Florianópolis, SC, Brasil; V Universidade Federal de Santa Catarina Departamento de Saúde Pública Florianópolis SC Brasil Universidade Federal de Santa Catarina. Departamento de Saúde Pública. Florianópolis, SC, Brasil

**Keywords:** Aged, Hearing Loss, Presbycusis, Depression, Diagnostic Self Evaluation, Health Surveys

## Abstract

**OBJECTIVE:**

To estimate the association between negative self-perception of hearing and depression in older adults in Southern Brazil.

**METHODS:**

This is a cross-sectional study conducted with data from the third wave of the EpiFloripa Idoso 2017/19 study, a population-based cohort of older adults (60+). A total of 1,335 older adults participated in this wave. The dependent variable was self-reported depression, and the main exposure was self-perception of hearing (negative; positive). For both the crude (bivariate) and adjusted analysis, the odds ratio (OR) was used as a measure of association and estimated by means of binary logistic regression analysis. The exposure variable was adjusted by sociodemographic and health covariates. A p value < 0.05 was adopted as statistically significant.

**RESULTS:**

The prevalence of negative self-perception of hearing and depression was 26.0% and 21.8%, respectively. In the adjusted analysis, the older adults with negative self-perception of hearing were 1.96 times more likely to report depression when compared to the ones with positive self-perception of hearing (p = 0.002).

**CONCLUSION:**

The association between negative self-perception of hearing and depression reflects the importance of reviewing health care actions for older adults, incorporating hearing-related issues, to ensure comprehensive care for this growing segment of the population.

## INTRODUCTION

The increase in life expectancy and the number of older adults in the population represents of late an important challenge for health policies regarding the guarantee of comprehensive care and the maintenance of autonomy and functional capacity of older adults^1^. The coexistence of multiple morbidities affects not only the risk of loss of functionality and quality of life, but also on the increase of healthcare expenses^2^.

Among the most prevalent morbidities with aging, age-related hearing loss (ARHL) stands out. It is progressive and bilateral, compromising speech intelligibility, having a negative impact on social engagement and cognitive skills in the elderly, representing risks of cognitive decline, social isolation and depression, thus being an important risk factor for healthy aging^3 , 4^.

On the other hand, depression is one of the most common mental disorders in the world, affecting about 350 million people and being regarded as a chronic disease^5^. Estimates indicate that this disorder will represent the greatest global burden of disease by 2030, being more impactful in low-income countries due to the lack of diagnosis and treatment^6^.

Depression is associated with the loss of interest for previously pleasurable activities, lack of energy, and even suicidal thoughts^7^. Regarding aging, this scenario may be even more serious due to decline in bio-psycho-functional capabilities which, along with the worsening of pre-existing diseases and the negative perception of the aging process, may represent an important risk factor for the development of depression^8^.

Hearing loss and depression are prevalent morbidities in older adults, and studies have tried to identify psychosocial and health mechanisms^9 , 10^ —as well as neuropathological ones —associated with the perception of hearing and mood regulation to enhance the planning of actions aimed at improving the quality of life of this population segment^11^.

Some studies have investigated the association between hearing loss and depression in population samples of older adults. However, the results are conflicting. While some studies have reported significant associations^12^, others failed to find them^17^.

The new demands generated by aging lead to the emerging need for actions geared towards prevention, diagnosis, and early treatment for comorbidities affecting older adults, in order to adapt the healthcare system to these changes, especially in low-income countries^1^.

Therefore, this study sought to investigate the association between negative self-perception of hearing and depression in older adult’s participants in a population-based study in Southern Brazil.

## METHODS

### Study Design and Location

This is a cross-sectional analysis conducted with data from the study *EpiFloripa Idoso: condições de saúde de idosos,* a home-based cohort with older adults (60 years or older) living in the urban area of the municipality of Florianópolis (SC), whose main objective was to investigate the living and health conditions of this population. The research has had two previous waves: the first started in 2009/2010, in which 1,705 older adults from the municipality were interviewed, and the second in 2013/2014 (totaling 1,197 people). The third wave took place between 2017 and 2019, involving 1,335 older adults (epifloripaidoso.paginas.ufsc.br).

Detailed procedures on sample planning, operational aspects, and strategies used in the first two waves have been previously described^20^. In this study, data from the third wave of the survey (2017-2019) were used, since it was the one to originate the investigation on the auditory aspects of the participants.

### Sampling and Data Collection Procedure

The sampling plan for 2017-2019 was assembled based on the sampling processes conducted in previous waves and on the data from the Census conducted by the Brazilian Institute of Geography and Statistics (IBGE) in 2010, in order to maintain the representativeness of the population of older adults of Florianopolis. From the third wave, the study became an open cohort, i.e., new older adults were included.

For the follow-up of the participants of the previous waves, the registration data were updated by telephone contact and the survey of deaths with the aid of data from the Mortality Information System (SIM). For the insertion of new subjects, the sample size was reassessed based on the older adult population of the municipality referring to the 2010 Census – 48,423 inhabitants. The parameters previously used for the prevalence calculation were maintained, with simple causal sample added to a value related to the effect of the estimated design — sample by conglomerates — of a proportion of expected losses and control for confounding factors. Considering the selection of the conglomerate sample, the same sectors of the baseline were maintained as the first stage unit. However, it was necessary to update the second-stage unit: the households. The number of households in each sector (census) was updated by the research technical team, since the most recent Census took place in 2010, following the same procedures. The data collection instrument, structured as a questionnaire and applied in a face-to-face interview format by trained interviewers, was assembled with the collaboration of the research technical team. The team held weekly meetings to update the questionnaire to be applied in the third wave. Priority was given to maintaining most of the questions applied in 2013 and 2014 because it is a longitudinal study. The content, clarity, and adequacy of the interview time were checked by supervisors and interviewers, and the questionnaire was applied with older adult’s non-participants of the research.

### Outcome

The dependent variable of this study was self-reported depression (no; yes), collected by the question: “Has a doctor or health professional ever said that you have or have had depression?”

### Exposure

The main exposure variable was self-perception of hearing (positive; negative), collected using the question: “In general, would you say that your hearing is: (a) excellent, (b) very good, (c) good, (d) fair, (e) poor, or (f) very poor?”. For those who used hearing aids, the questioning referred to the quality of hearing during the use of the device. In this study, the categories “excellent,” “very good,” and “good” were considered as positive self-perception of hearing, and the categories “regular,” “bad,” and “very bad” as negative self-perception of hearing.

### Covariates

The covariates in this study referred to the sociodemographic characteristics and health status of the individuals: sex (male; female); self-reported skin color (white; brown/yellow/indigenous; black); age group (60 to 69; 70 to 79; 80 years or older); schooling level (0 to 8; 9 to 11; 12 years or older); self-reported comorbidities (Diabetes Mellitus (DM) (no; yes), systemic arterial hypertension (SAH) (no; yes), stroke (no; yes), and use of hearing aid (no; yes, in one ear; yes, in both ears)); physical condition of the participant (bedridden; wheelchair user; ambulant).

### Data Analysis

To describe the categorical variables of the sample, data were represented by absolute and relative frequencies, with their respective 95% confidence intervals (95%CI). The prevalence of the outcome (%) was estimated for all variables of the study.

For both the crude (bivariate) and adjusted analysis, the OR was used as a measure of association, estimated by means of the binary logistic regression analysis. The main exposure variable was adjusted by all study covariates, regardless of p value, to evaluate the effect of all exposure variables on the outcome. Variables were included simultaneously in the adjusted analysis and a 5% significance level was adopted.

Data analysis was conducted in the Stata program version 14.0, considering the study design and the sample weight of the database (svy command).

### Ethical Aspects

The EpiFloripa Idoso 2017/2019 study was approved as an amendment of the previous study by the Committee for Ethics in Research with Human Beings (CEPSH) under number 1,957,977 on March 9, 2017. All the subjects who agreed to participate in the research provided an informed consent form.

## RESULTS

Most participants (63.7%) were female, aged from 70 to 79 years (43.6%), White (88.3%), and with lower schooling level (54.9%). The prevalence of depression reported was 21.8% and the prevalence of negative self-perception of hearing was 26.0%. Regarding morbidities, 59.5% reported hypertension, 25.7% diabetes, and 9.5% stroke. The use of hearing aids was reported by 7.5% of the sample and most older adults were ambulant (98.1%) ( Table 1 ).

**Table 1:** Descriptive analysis of the sample characteristics and prevalence of depression according to study variables. EpiFloripa study 2017–2019. Florianópolis, Santa Catarina (SC), Brazil (n = 1,335).

Variable	n	Percentage of total sample%^a^	95%CI	Prevalence of depression%
Gender (n = 1.335)				
Men	510	36.3	33.0–39.5	14.3
Women	825	63.7	60.4–66.9	27.5
Skin color (n = 1,334)				
White	1,172	88.3	84.6–91.1	22.0
Mixed-race/Yellow/Indigenous	99	7.6	5.7–9.9	25.3
Black	63	4.1	2.7–6.2	27.0
Age group (years) (n = 1,335)				
60–69	461	30.2	25.3–35.5	25.2
70–79	554	43.6	39.1–48.2	21.6
≥ 80	320	26.2	22.3–30.2	20.0
Schooling level (years) (n = 1,329)				
0–8	715	54.9	48.3–61.4	25.3
9–11	215	15.8	12.8–19.3	23.7
≥ 12	399	29.3	24.4–34.6	16.3
Diabetes (n = 1,335)				
No	999	74.3	70.5–77.7	20.8
Yes	336	25.7	22.2–29.4	27.4
Hypertension (n = 1,335)				
No	516	40.5	35.7–45.4	17.8
Yes	819	59.5	54.5–64.2	25.4
Stroke (n = 1,335)				
No	1,195	90.5	88.1–92.2	21.7
Yes	140	9.5	7.7–11.8	29.3
Use of hearing aid (n = 1,335)				
No	1,240	92.5	90.1–94.2	22.3
Yes, in one ear	42	3.6	2.4–6.3	26.2
Yes, in both ears	53	3.9	2.5–5.2	24.5
Physical condition (n = 1,335)				
Bedridden	18	1.3	0.7–2.4	33.3
Wheelchair user	11	0.6	0.3–1.2	36.4
Ambulant	1,306	98.1	96.9–98.8	22.2
Self-perception of hearing (n = 1,287)				
Positive	947	74.0	70.3–77.4	19.2
Negative	335	26.0	22.5–29.7	29.9

95%CI: 95% confidence interval

^a^ Weighted percentage.

The prevalence of depression was higher among women (27.5%), Blacks (27.0%), and people in the younger age group (25.2%). We observed a higher prevalence of depression in older people with negative self-perception of hearing (29.9%) when compared to those with positive self-perception (19.2%). Subjects with lower schooling levels had a higher prevalence of depression (25.3%). Depression was also more prevalent in subjects with comorbidities —diabetes, hypertension, and history of stroke —and among wheelchair users (36.4%) ( Table 1 ).

In the crude analysis, older adults with negative self-perception of hearing were 1.58 times more likely (95%CI: 1.10–2,29) to report depression when compared to those with positive self-perception of hearing ( Table 2 ).

**Table 2:** Crude analysis of the association between self-perception of hearing and other covariates with the outcome of the study (depression). EpiFloripa study 2017/2019. Florianópolis, Santa Catarina (SC), Brazil.

Variable	Depression	p

	Crude OR^a^ (95%CI)	
Gender		
Men	Ref.	< 0.001
Women	2.34 (1.46–3.77)	
Skin color		0.608
White	Ref.	
Mixed-race/Yellow/Indigenous	1.04 (0.60–1.82)	
Black	1.25 (0.53–2.93)	
Age group (years)		0.025
60–69	Ref.	
70–79	0.59 (0.37–0.95)	
≥ 80	0.56 (0.34–0.93)	
Schooling level (years)		0.147
0–8	Ref.	
9–11	0.63 (0.38–1.04)	
≥ 12	0.71 (0.42–1.19)	
Diabetes		0.563
No	Ref.	
Yes	1.15 (0.70–1.88)	
Hypertension		0.086
No	Ref	
Yes	1.34 (0.95–1.90)	
Stroke		0.102
No	Ref.	
Yes	1.55 (0.91–2.65)	
Use of hearing aid		0.787
No	Ref.	
Yes, in one ear	1.32 (0.32–5.36)	
Yes, in both ears	0.99 (0.46–2.11)	
Physical condition		0.073
Bedridden	Ref.	
Wheelchair user	0.28 (0.43–1.90)	
Ambulant	0.26 (0.64–1.08)	
Self-perception of hearing		0.013
Positive	Ref.	
Negative	1.58 (1.10–2.29)	

OR: *odds ratio* ; Ref.: reference; 95%CI: 95% confidence interval.

^a ^Crude logistic regression.

In the analysis adjusted for sociodemographic covariates and health conditions, the association remained with a odds ratio almost twice as high (OR = 1.96; IC95%: 1.30–2.97) ( Table 3 ).

**Table 3:** Adjusted analysis of the association between self-perception of hearing and depression. EpiFloripa study 2017/2019. Florianópolis, Santa Catarina (SC), Brazil.

Depression		

Self-perception of hearing	Adjusted OR^a^ (95%CI)	p
Positive	Ref.	0.002
Negative	1.96 (1.30–2.97)	

OR: *odds ratio* ; Ref.: reference; 95%CI: 95% confidence interval.

^a^ Analysis adjusted for gender, skin color, age group, schooling level, Diabetes Mellitus, systemic arterial hypertension, stroke, use of hearing aid, and physical condition.

## DISCUSSION

This study, conducted with older adults in Southern Brazil, investigated the association between negative self-perception of hearing and depression. Our findings showed that subjects with a negative self-perception of hearing were almost twice as likely to report depression compared to those with a positive self-perception.

In the crude analysis, we highlight the higher prevalence of depression in women, of low schooling level, and with chronic diseases, corroborating the findings of a previous study and stressing the importance of using these covariates in the adjustments of the other analyses to minimize their influence^15^. The other covariates —schooling level, skin color, and age group — which also showed differences between the subgroups, were included in the adjustments, since their possible influence has already been described in the literature^9 , 21^.

Studies have evaluated self-reported depression in older adults and found a prevalence that varied from 24% to 30.6%^22 , 23^. As previously mentioned, this variation in prevalence is possibly related to methodological differences among these studies —study design, classification criteria, and adjustment covariates —and with the different populations studied^9 , 24^.

The most commonly used indicator of hearing loss in Brazilian population studies is self-reported hearing loss, investigated by using questions related to the perception of hearing difficulty and hearing assessment. The prevalence of self-reported hearing loss in older adults obtained in previous studies ranged from 5.2% to 30.4%^25 , 26^, corroborating our findings. Hearing loss affects more than 30% of adults over 50 years, and the prevalence almost doubles the more decades of life the individual accumulates^27^. Moreover, most people with hearing loss remain undiagnosed and untreated for many years^3 , 28^.

For this reason, the negative self-perception of hearing by older adults should be used as an important indicator, since hearing loss may be associated with adverse health outcomes during the aging process, including anxiety and depression^4 , 8^, as well as it may generate communication difficulties and compromise social functioning^29^.

Although some studies show an association between hearing loss and depression, the underlying etiological mechanisms have not yet been well established. As already mentioned, hearing loss can lead to isolation and frustration, especially when communication is compromised. The social impact, the reduction in quality of life, and the economic burden for the patient and the family have also been described as having an influence on this relation^28^. The plasticity resulting from hearing loss, which affects the most central regions of the cortex, make it likely that neurophysiological mechanisms also participate in this process. However, future clinical and experimental studies should clarify these links and mechanisms^15^.

The association between negative perception of hearing and depression found in this study may be linked to the fact that hearing loss affects the communication process, contributing to a process of isolation and to the development of depressive conditions, agreeing with studies that found an association between hearing loss and depression^12 -[Bibr B13][Bibr B14][Bibr B15] 16^.

Recent studies have shown that hearing rehabilitation, including the use of sound amplification devices, helps improve mental health, but evidence is still scarce, and the subject needs further studies^24^.

Some elements should be considered when interpreting the results of this study. The use of self-reported measures may be considered a limitation since prevalence may be under or overestimated. However, studies showed that self-reported hearing loss, especially among older adults, has validity in population studies with reliable sensitivity and specificity values^30^. We also highlight the possible reverse causality bias, inherent to cross-sectional analyses.

As a potentiality, we highlight the fact that the study is of interest in the context of population aging and involves prevalent morbidities. Moreover, the insertion of auditory data in the *Epifloripa idoso* study represents an important uniqueness since it is a representative study of the Florianópolis population and allows a future longitudinal analysis of these subjects from the perspective of integrality of care for healthy aging.

Thus, the association found between the negative self-perception of hearing and the report of depression alerts to the importance of investigating these morbidities in the context of aging. Hearing loss should not be regarded as a natural aging process without perspectives for treatment and rehabilitation, since, besides being prevalent, it is a risk factor for several negative health outcomes, including negative impacts on mental and physical health of older adults. The social engagement and participation of older adults may be protective factors of several adverse outcomes. Thus, we highlight the importance of developing preventive techniques in primary care and expanding the assessment, diagnosis, and access to rehabilitation services that make up the planning agenda for support to older adults at all points of the health care network, ensuring the principle of integrality in health care for this population.
