# Supplementary material for: Negative self-perception of hearing and depression in older adults: a population-based study
Source: Rev Saude Publica. 2023 Mar 29;57:15. doi: 10.11606/s1518-8787.2023057004675 (PMC10118410; doi:10.11606/s1518-8787.2023057004675)
Supplement: Translation

# Translation: Autopercepção negativa da audição e depressão em idosos: um estudo de base populacional

**Authors:** Karina Mary de Paiva, Alessandra Giannella Samelli, Pamela Lopes de Oliveira, Danúbia Hillesheim, Patrícia Haas, Paulo Adão de Medeiros, Eleonora d’Orsi

**Affiliations:** I Brasil Universidade Federal de Santa Catarina. Departamento de Fonoaudiologia. Florianópolis, SC, Brasil; II Brasil Universidade de São Paulo. Faculdade de Medicina. Departamento de Fisioterapia, Fonoaudiologia e Terapia Ocupacional. São Paulo, SP, Brasil; III Brasil Universidade Federal de Santa Catarina. Programa de Pós-Graduação em Saúde Coletiva. Florianópolis, SC, Brasil; IV Brasil Associação Nacional de Gerontologia. Florianópolis, SC, Brasil; V Brasil Universidade Federal de Santa Catarina. Departamento de Saúde Pública. Florianópolis, SC, Brasil

**Keywords:** Idoso, Perda Auditiva, Presbiacusia, Depressão, Autoavaliação Diagnóstica, Inquéritos Epidemiológicos

## Abstract

**OBJETIVO:**

Estimar a associação entre a autopercepção negativa da audição e a depressão em idosos do sul do Brasil.

**MÉTODOS:**

Trata-se de um estudo transversal realizado com dados da terceira onda do estudo EpiFloripa Idoso 2017/19, de coorte de base populacional de idosos (60+). Participaram desta onda 1.335 idosos. A variável dependente foi a depressão autorreferida e a exposição principal foi a autopercepção auditiva (negativa; positiva). Tanto para a análise bruta (bivariada) quanto para a ajustada, a odds ratio (OR) foi utilizada como medida de associação e estimada por meio da análise de Regressão Logística Binária. A variável de exposição foi ajustada pelas covariáveis sociodemográficas e de saúde. Adotou-se o valor de p < 0,05 como estatisticamente significativo.

**RESULTADOS:**

A prevalência da autopercepção negativa da audição e depressão foi de 26,0% e 21,8%, respectivamente. Na análise ajustada, idosos com autopercepção negativa da audição apresentaram 1,96 vezes mais chance de referirem depressão quando comparados aos idosos com autopercepção positiva da audição (p = 0,002).

**CONCLUSÃO:**

A associação encontrada entre a autopercepção negativa auditiva e a depressão reflete a importância de rever as ações de atenção à saúde do idoso, incorporando questões relacionadas à audição para a garantia da atenção integral a esta parcela crescente da população.

## INTRODUÇÃO

O aumento da expectativa de vida e do número de idosos na população tem representado um importante desafio para as políticas de saúde no que se refere à garantia da integralidade do cuidado e da manutenção da autonomia e da capacidade funcional dos idosos^1^. A coexistência de múltiplas morbidades reflete não somente no risco de perda de funcionalidade e de qualidade de vida, mas também no aumento dos gastos em saúde^2^.

Dentre as morbidades mais prevalentes com o envelhecimento, destaca-se a *age-related hearing loss* (ARHL), de caráter progressivo e bilateral, que compromete a inteligibilidade de fala, impactando negativamente no engajamento social e nas habilidades cognitivas do idoso, representando riscos de declínio cognitivo, isolamento social e depressão, sendo, portanto, um importante fator de risco ao envelhecimento saudável^3 , 4^.

Por outro lado, a depressão é um dos transtornos mentais mais frequentes no mundo, acometendo cerca de 350 milhões de pessoas e sendo considerada uma doença crônica^5^. Estimativas apontam que este transtorno representará a maior carga global de doenças até 2030, sendo mais impactante em países de baixa renda devido à falta de diagnóstico e tratamento^6^.

A depressão apresenta-se associada à perda de interesse por atividades anteriormente prazerosas, à falta de energia e, até mesmo, aos pensamentos suicidas^7^. No contexto do envelhecimento, este quadro pode ser ainda mais grave em função do declínio das capacidades biopsicofuncionais, que, aliado ao agravamento de morbidades preexistentes e à percepção negativa do processo do envelhecimento, pode representar um importante fator de risco para o desenvolvimento da depressão^8^.

A perda auditiva e a depressão são morbidades prevalentes nos idosos e estudos tentam identificar mecanismos psicossociais e de saúde^9 , 10^– assim como neuropatológicos – associados à percepção da audição e da regulação do humor a fim de potencializar o planejamento de ações voltadas à qualidade de vida desse segmento populacional^11^.

Alguns estudos vêm investigando a associação entre a perda auditiva e a depressão em amostras populacionais de idosos. No entanto, os resultados são conflitantes. Alguns estudos relataram associações significativas^12^, enquanto outros não verificaram tal associação^17^.

As novas demandas geradas pelo envelhecimento remetem à necessidade emergente de ações voltadas à prevenção, ao diagnóstico e ao tratamento precoce para as comorbidades que acometem o idoso para, assim, adequar o sistema de saúde frente a essas mudanças, especialmente nos países de baixa renda^1^.

Diante disso, o objetivo deste estudo foi investigar a associação entre a autopercepção negativa da audição e a depressão em idosos participantes de um estudo de base populacional no sul do Brasil.

## MÉTODOS

### Delineamento e Local do Estudo

Trata-se de uma análise transversal realizada com dados do estudo *EpiFloripa Idoso: condições de saúde de idosos* e de coorte de base domiciliar com idosos (60 anos ou mais) residentes na zona urbana do município de Florianópolis (SC), cujo objetivo principal foi investigar as condições de vida e saúde da população idosa. A pesquisa já contou com duas ondas anteriores: a primeira iniciou em 2009/2010, na qual foram entrevistados 1.705 idosos da cidade, e a segunda em 2013/2014 (1.197 idosos). A terceira onda se realizou entre 2017 e 2019, envolvendo 1.335 idosos (epifloripaidoso.paginas.ufsc.br).

Procedimentos detalhados sobre o planejamento amostral, aspectos operacionais e estratégias utilizadas nas duas primeiras ondas foram descritos previamente^20^. Neste estudo, foram utilizados os dados da terceira onda da pesquisa (2017–2019), pois foi a partir desta que se iniciou a investigação sobre os aspectos auditivos dos participantes.

### Procedimento de Amostragem e Coleta de Dados

O plano amostral de 2017–2019 foi construído com base nos processos de amostragem realizados nas ondas anteriores e nos dados do Censo realizado pelo Instituto Brasileiro de Geografia e Pesquisa (IBGE) de 2010, com a finalidade de manter a representatividade da população idosa de Florianópolis. A partir da terceira onda, o estudo tornou-se uma coorte aberta, ou seja, novos idosos foram incorporados.

Para o acompanhamento dos idosos participantes das ondas anteriores, realizou-se a atualização dos dados cadastrais via contato telefônico e levantamento dos óbitos com auxílio dos dados do Sistema de Informações de Mortalidade (SIM). Para a inserção de novos idosos, foi reavaliado o tamanho da amostra com base na população idosa do município referente ao Censo de 2010 – 48.423 habitantes. Mantiveram-se os parâmetros usados anteriormente para o cálculo de prevalência, com amostra causal simples adicionada de um valor relativo ao efeito do delineamento estimado – amostra por conglomerados – de uma proporção de perdas previstas e controle para fatores de confusão. Considerando a seleção da amostra por conglomerados, foram mantidos como unidade de primeiro estágio os mesmos setores da linha de base. Contudo, foi necessário atualizar a unidade de segundo estágio: os domicílios. O número de domicílios em cada setor (arrolamento) foi atualizado pela equipe técnica da pesquisa, uma vez que o Censo mais recente ocorreu em 2010, seguindo-se os mesmos procedimentos. O instrumento de coleta de dados, estruturado por meio de um questionário e aplicado em formato de entrevista presencial por entrevistadores treinados, foi construído com a colaboração da equipe técnica da pesquisa. A equipe realizou encontros semanais para atualização do questionário a ser aplicado na terceira onda. Priorizou-se a manutenção da maioria das questões aplicadas em 2013 e 2014 por se tratar de um estudo longitudinal. O conteúdo, a clareza e a adequação do tempo de entrevista foram verificados pelos supervisores e entrevistadores, sendo aplicado o questionário com idosos não participantes da pesquisa.

### Desfecho

A variável dependente deste estudo foi a depressão autorreferida (não; sim), coletada por meio da pergunta: *“* Algum médico ou profissional de saúde já disse que o(a) Sr.(a.) tem/teve depressão?”.

### Exposição

A variável de exposição principal foi a autopercepção auditiva (positiva; negativa), coletada por meio da pergunta: *“* Em geral, o(a) Sr.(a.) diria que sua audição é: (a) excelente, (b) muito boa, (c) boa, (d) regular, (e) ruim ou (f) muito ruim?”. Para aqueles que usavam aparelho auditivo, o questionamento se referia à qualidade da audição durante o uso do aparelho. Neste estudo, as categorias “excelente”, “muito boa” e “boa” foram consideradas como autopercepção positiva da audição, e as categorias “regular”, “ruim” e “muito ruim” como autopercepção negativa da audição.

### Covariáveis

As covariáveis deste estudo foram referentes às características sociodemográficas e à situação de saúde dos indivíduos: sexo (masculino; feminino); cor da pele autorreferida (branca; parda/amarela/indígena; negra ou preta); faixa etária (60 a 69; 70 a 79; 80 anos ou mais); escolaridade (0 a 8 anos; 9 a 11 anos; 12 anos ou mais); comorbidades autorreferidas (diabetes mellitus (DM) (não; sim), hipertensão arterial sistêmica (HAS) (não; sim), acidente vascular cerebral (não; sim) e uso de aparelho auditivo (não; sim, em uma orelha; sim, nas duas orelhas)); condição física do participante (acamado; cadeirante; deambulante).

### Análise dos Dados

Para a descrição das variáveis categóricas da amostra, os dados foram representados por frequências absolutas e relativas, com seus respectivos Intervalos de 95% de Confiança (IC95%). Foram estimadas as prevalências do desfecho (%) para todas as variáveis do estudo.

Tanto para a análise bruta (bivariada) quanto para a ajustada, a OR foi utilizada como medida de associação, estimada por meio da análise de Regressão Logística Binária. A variável de exposição principal foi ajustada por todas as covariáveis do estudo, independentemente do valor de p, com o intuito de avaliar o efeito de todas as variáveis de exposição sobre o desfecho. As variáveis foram incluídas de forma simultânea na análise ajustada e adotou-se o nível de significância de 5%.

A análise dos dados foi conduzida no software Stata versão 14.0, considerando-se o delineamento do estudo e o peso amostral do banco de dados ( *comando svy* ).

### Aspectos éticos

O estudo EpiFloripa Idoso 2017/2019 foi aprovado como uma emenda do estudo anterior pelo Comitê de Ética em Pesquisa com Seres Humanos (CEPSH), sob número 1.957.977, em 9 de março de 2017. Todos os idosos que aceitaram participar da pesquisa assinaram um termo de consentimento livre e esclarecido.

## RESULTADOS

A maioria dos participantes (63,7%) era do sexo feminino, faixa etária entre 70 e 79 anos de idade (43,6%), pele branca (88,3%) e com menor nível de escolaridade (54,9%). A prevalência de depressão relatada pelos idosos foi igual a 21,8%, enquanto a da autopercepção negativa da audição foi de 26,0%. Com relação às morbidades, 59,5% dos idosos relataram hipertensão arterial, 25,7% diabetes e 9,5% acidente vascular cerebral (AVC). O uso de aparelhos auditivos foi relatado por 7,5% da amostra e a maioria dos idosos era deambulante (98,1%) ( Tabela 1 ).

**Tabela 1:** Análise descritiva das características da amostra e prevalência de depressão segundo variáveis do estudo. Estudo EpiFloripa 2017–2019. Florianópolis, Santa Catarina (SC), Brasil (n = 1.335).

Variável	n	Percentual da amostra total%^a^	IC95%	Prevalência de depressão%
Sexo (n = 1.335)				
Masculino	510	36,3	33,0–39,5	14,3
Feminino	825	63,7	60,4–66,9	27,5
Cor da pele (n = 1.334)				
Branca	1.172	88,3	84,6–91,1	22,0
Parda/amarela/indígena	99	7,6	5,7–9,9	25,3
Negra ou preta	63	4,1	2,7–6,2	27,0
Faixa etária (anos) (n = 1.335)				
60–69	461	30,2	25,3–35,5	25,2
70–79	554	43,6	39,1–48,2	21,6
≥ 80	320	26,2	22,3–30,2	20,0
Escolaridade (anos) (n = 1.329)				
0–8	715	54,9	48,3–61,4	25,3
9–11	215	15,8	12,8–19,3	23,7
≥ 12	399	29,3	24,4–34,6	16,3
Diabetes (n = 1.335)				
Não	999	74,3	70,5–77,7	20,8
Sim	336	25,7	22,2–29,4	27,4
Hipertensão (n = 1.335)				
Não	516	40,5	35,7–45,4	17,8
Sim	819	59,5	54,5–64,2	25,4
AVC (n = 1.335)				
Não	1.195	90,5	88,1–92,2	21,7
Sim	140	9,5	7,7–11,8	29,3
Uso de aparelho auditivo (n = 1.335)				
Não	1.240	92,5	90,1–94,2	22,3
Sim, em uma orelha	42	3,6	2,4–6,3	26,2
Sim, nas duas orelhas	53	3,9	2,5–5,2	24,5
Condição física (n = 1.335)				
Acamado	18	1,3	0,7–2,4	33,3
Cadeirante	11	0,6	0,3–1,2	36,4
Deambulante	1.306	98,1	96,9–98,8	22,2
Autopercepção auditiva (n = 1.287)				
Positiva	947	74,0	70,3–77,4	19,2
Negativa	335	26,0	22,5–29,7	29,9

IC95%: intervalo de 95% de confiança.

^a^Percentual ponderado.

A prevalência de depressão foi maior entre mulheres (27,5%), negros/pretos (27,0%), na faixa etária mais jovem (25,2%). Maior prevalência de depressão foi observada em idosos com autopercepção negativa da audição (29,9%) quando comparados aos idosos com autopercepção positiva (19,2%). Observou-se que idosos com menor nível de escolaridade apresentaram maior prevalência de depressão (25,3%). A depressão foi mais prevalente também em idosos com comorbidades – diabetes, hipertensão e histórico de AVC – e entre os indivíduos cadeirantes (36,4%) ( Tabela 1 ).

Na análise bruta, idosos com autopercepção negativa da audição apresentaram 1,58 vezes mais chance (IC95%: 1,10–2,29) de relatarem depressão quando comparados aos idosos com autopercepção positiva da audição ( Tabela 2 ).

**Tabela 2:** Análise bruta da associação entre autopercepção auditiva e demais covariáveis com o desfecho do estudo (depressão). Estudo EpiFloripa 2017/2019. Florianópolis, Santa Catarina (SC), Brasil.

Variável	Depressão	p

	OR bruta^a^(IC95%)	
Sexo		< 0,001
Masculino	Ref.	
Feminino	2,34 (1,46–3,77)	
Cor da pele		
Branca	Ref.	0,608
Parda/amarela/indígena	1,04 (0,60–1,82)	
Negra ou preta	1,25 (0,53–2,93)	
Faixa etária (anos)		0,025
60–69	Ref.	
70–79	0,59 (0,37–0,95)	
≥ 80	0,56 (0,34–0,93)	
Escolaridade (anos)		0,147
0–8	Ref.	
9–11	0,63 (0,38–1,04)	
≥ 12	0,71 (0,42–1,19)	
Diabetes		0,563
Não	Ref.	
Sim	1,15 (0,70–1,88)	
Hipertensão		0,086
Não	Ref.	
Sim	1,34 (0,95–1,90)	
AVC		0,102
Não	Ref.	
Sim	1,55 (0,91–2,65)	
Uso de aparelho auditivo		0,787
Não	Ref.	
Sim, em uma orelha	1,32 (0,32–5,36)	
Sim, nas duas orelhas	0,99 (0,46–2,11)	
Condição física		0,073
Acamado	Ref.	
Cadeirante	0,28 (0,43–1,90)	
Deambulante	0,26 (0,64–1,08)	
Autopercepção auditiva		0,013
Positiva	Ref.	
Negativa	1,58 (1,10–2,29)	

OR: *odds ratio* ; Ref.: referência; IC95%: intervalo de 95% de confiança.

^a^Regressão logística bruta.

Na análise ajustada pelas covariáveis sóciodemográficas e condições de saúde, a associação manteve-se com uma razão de chances quase duas vezes maior (OR = 1,96; IC95%: 1,30–2,97) ( Tabela 3 ).

**Tabela 3:** Análise ajustada da associação entre autopercepção auditiva e depressão. Estudo EpiFloripa 2017/2019. Florianópolis, Santa Catarina (SC), Brasil.

Depressão		

Autopercepção auditiva	OR ajustada^a^(IC95%)	p
Positiva	Ref.	0,002
Negativa	1,96 (1,30–2,97)	

OR: *odds ratio* ; Ref.: referência; IC95%: intervalo de 95% de confiança.

^a^Análise ajustada por sexo, cor da pele, faixa etária, escolaridade, diabetes mellitus, hipertensão arterial sistêmica, acidente vascular cerebral, uso de aparelho auditivo e condição física.

## DISCUSSÃO

Este estudo, realizado com idosos na região sul do Brasil, investigou a associação entre autopercepção negativa da audição e depressão. Nossos achados demonstraram que idosos com autopercepção negativa da audição apresentaram quase o dobro de chance de relatarem ter depressão se comparados aos idosos com autopercepção positiva.

Na análise bruta, destaca-se a maior prevalência de depressão em idosos do sexo feminino, baixa escolaridade e com as doenças crônicas, corroborando os achados de estudo prévio e ressaltando a importância de utilizar essas covariáveis nos ajustes das demais análises para minimizar a sua influência^15^. As demais covariáveis – escolaridade, raça/etnia e faixa-etária –, que também mostraram diferenças entre os subgrupos, foram incluídas nos ajustes, uma vez que já foi descrito na literatura a possível influência dessas variáveis sobre a depressão^9 , 21^.

Estudos avaliaram a depressão autorreferida por idosos e encontraram uma prevalência que variou de 24% a 30,6%^22 , 23^. Conforme já mencionado, esta variação na prevalência está relacionada, possivelmente, com as diferenças metodológicas – desenho do estudo, critérios de classificação e covariáveis de ajuste – entre os estudos e com as diferentes populações estudadas^9 , 24^.

O indicador de perda auditiva mais usado em estudos populacionais brasileiros é a perda auditiva autorreferida, investigada por meio do uso de perguntas relacionadas à percepção da dificuldade auditiva e da avaliação da audição. A prevalência da perda auditiva autorreferida por idosos obtida em estudos prévios variou entre 5,2% a 30,4%^25 , 26^, corroborando nossos achados. Sabe-se, ainda, que a perda auditiva afeta mais de 30% dos adultos acima de 50 anos, sendo que a prevalência quase dobra conforme mais décadas de vida o indivíduo acumula^27^. Além disso, a maioria das pessoas com perda auditiva permanece sem diagnóstico e tratamento por muitos anos^3 , 28^.

Por este motivo, a autopercepção negativa da audição por idosos deve ser utilizada como um importante indicador, uma vez que a perda auditiva pode estar associada a desfechos adversos em saúde durante o processo de envelhecimento, incluindo a ansiedade e a depressão^4 , 8^, bem como pode gerar dificuldades de comunicação e comprometer o funcionamento social^29^.

Apesar de alguns estudos mostrarem que existe uma associação entre a perda auditiva e a depressão, os mecanismos etiológicos subjacentes ainda não foram bem estabelecidos. Conforme já mencionado, a perda auditiva pode levar ao isolamento e à frustração, principalmente quando a comunicação fica comprometida. O impacto social, a diminuição na qualidade de vida e a carga econômica para o paciente e para a família também já foram descritos como tendo influência nesta relação^28^. A plasticidade decorrente da perda auditiva, que atinge as regiões mais centrais do córtex, não torna improvável que mecanismos neurofisiológicos também participem deste processo. No entanto, estudos clínicos e experimentais futuros devem esclarecer essas redes e mecanismos^15^.

A associação verificada neste estudo entre percepção negativa da audição e depressão pode estar atrelada ao fato de a perda auditiva comprometer o processo de comunicação, contribuindo para um processo de isolamento e para o desenvolvimento de quadros depressivos, o que vai de acordo com estudos que verificaram associação entre perda auditiva e depressão^12^.

Estudos recentes vêm mostrando que a reabilitação auditiva, incluindo o uso de dispositivos de amplificação sonora, auxilia na melhora da saúde mental, mas as evidências ainda são escassas e o tema necessita de estudos adicionais^24^.

Destacam-se alguns elementos que devem ser considerados ao interpretar os resultados deste estudo. O uso de medidas autorreferidas pode ser considerado uma limitação, visto que pode haver prevalência sub ou superestimada. Contudo, estudos apontaram que o autorrelato da perda auditiva, especialmente entre idosos, apresenta validade em estudos de nível populacional com valores de sensibilidade e especificidade confiáveis^30^. Também destaca-se o possível viés de causalidade reversa, inerente às análises transversais.

Como potencialidade, destaca-se o fato de o estudo envolver morbidades prevalentes e de interesse no contexto do envelhecimento populacional. Além disso, a inserção de dados auditivos no estudo *Epifloripa idoso* representa um importante diferencial, uma vez que se trata de um estudo representativo da população de Florianópolis e possibilita uma futura análise longitudinal desses idosos na perspectiva da integralidade do cuidado para um envelhecimento saudável.

Sendo assim, a associação encontrada entre a autopercepção negativa da audição e o relato de depressão alerta para a importância da investigação dessas morbidades no contexto do envelhecimento. A perda auditiva não pode ser considerada um processo natural do envelhecimento sem perspectivas para tratamento/reabilitação, pois, além de prevalente, é fator de risco para vários desfechos negativos em saúde, incluindo impactos negativos sobre a saúde mental e física do idoso. Sabe-se que o engajamento e a participação social dos idosos podem ser fatores protetores de vários desfechos adversos. Assim, destaca-se a importância de elaborar técnicas preventivas na atenção primária e ampliar a avaliação, o diagnóstico e o acesso aos serviços de reabilitação que compõem a agenda de planejamento para suporte ao idoso em todos os pontos da rede de atenção à saúde, garantindo o princípio da integralidade na atenção à saúde dessa população.
